# Trends in Glaucoma Medication Expenditures under Universal Health Coverage: A National Population-Based Longitudinal Survey in Taiwan

**DOI:** 10.1155/2015/243401

**Published:** 2015-06-02

**Authors:** Shin-Lin Chiu, Chiao-Lee Chu, Chih-Hsin Muo, Chiu-Liang Chen, Shou-Jen Lan

**Affiliations:** ^1^Department of Ophthalmology, Changhua Christian Hospital, 135 Nanxiao Street, Changhua City, Changhua County 500, Taiwan; ^2^Department of Healthcare Administration Management, Asia University, 500 Lioufeng Road, Wufeng, Taichung 41354, Taiwan; ^3^Management Office for Health Data, China Medical University Hospital, 2 Yude Road, Taichung 40447, Taiwan; ^4^Department of Orthopedics, Changhua Christian Hospital, 135 Nanxiao Street, Changhua City, Changhua County 500, Taiwan

## Abstract

Medical care in Taiwan is well known for its low cost, high efficiency, high quality, excellent medical accessibility, and high equity. We investigate the trends in medication expenditures for glaucoma from 1997 to 2010. The results show that higher medical expenditures were incurred by patients who were aged ≥40 years, male patients, and patients in the highest salary population whereas lower medical expenditures were incurred by blue-collar workers. The medications with the most significant increases in expenditure were prostaglandin analogs (PGAs), *α*-agonists, and fixed combinations, whereas the medications with the most significant decreases in expenditure were *β*-blockers and cholinergic agonists. The number of trabeculectomies shows two downward break points in 1999 and 2000 when PGAs were listed and were reimbursed. These results suggest socioeconomic disparities in glaucoma care, as well as understanding of the changes in the expenditure of glaucoma medications under such universal health insurance coverage system.

## 1. Introduction

Glaucoma is the second leading cause of blindness, affecting approximately 60.5 million people worldwide or about 2.6% of the population over the age of 40 [1]. Although the most prevalent form of glaucoma in western countries and in many other parts of the world is open-angle glaucoma (OAG) [1], angle-closure glaucoma (ACG) is the most common type of glaucoma among the Han Chinese [2].

Blindness due to glaucoma may be preventable if patients are given adequate treatment. Factors associated with the development of glaucoma include increased intraocular pressure (IOP), higher cup-to-disc ratio, aging, thinner central corneal thickness, family history of glaucoma, myopia, and chronic and systemic diseases such as diabetes [3, 4]. Most ophthalmologists treat glaucoma by lowering the IOP using one of three modalities—topical antiglaucoma drugs, laser treatment, or glaucoma surgery. Types of topical medication include *β*-blockers, *α*-agonists, prostaglandin analogs (PAGs), carbonic anhydrase inhibitors (CAIs), cholinergic agonists, and adrenergic agonists [5]. Because some patients require multiple daily dosing, fixed combination eye drops have been developed to enhance and reinforce patient compliance [6].

Recent studies have shown that glaucoma treatment in some developed countries causes a significant financial burden on the health care system [7–9]. In this study, we used the National Longitudinal Health Insurance Database 2000 (LHID2000) to examine trends in glaucoma medication expenditure in Taiwan from 1997 to 2010. The Taiwan National Health Insurance (NHI) program is a mandatory single-payer health insurance system under which all residents are covered. The NHI program has been in existence since 1995 and by the end of 2010 the coverage rate was 99% of Taiwan's population of 23.1 million. Health spending accounted for 6.9% of GDP in Taiwan. Medical care in Taiwan is well known for its low cost, convenience, high efficiency, high quality, and excellent medical accessibility [10–12]. The nationwide population-based dataset provides an opportunity to explore the trends in glaucoma medication expenditure.

## 2. Materials and Methods

### 2.1. Database

This study was designed as a population-wide retrospective review using the Taiwan National Health Insurance Research Database (NHIRD). The NHIRD contains a large number of computerized records including registration files, medication and treatment regimens, and information on surgery. Claim reimbursement data from patients since 1999 are provided to researchers in an electronically encrypted form. Data contained in the LHID2000 are randomly selected from one million subjects from the NHIRD and are made available for research purposes [13]. Data from the LHID2000 used in this study included patient identification numbers, gender and age, monthly salary, occupation type, diagnostic data, antiglaucoma drug codes, and surgery codes. Diseases are defined in accordance with A codes (A230) before 2000 and International Classification of Diseases, 9th Revision, Clinical Modification (ICD-9-CM365), after 2000.

The study received approval from the Ethics Committee of the Institutional Review Board of the Changhua Christian Hospital and was conducted in accordance with the tenants of the Declaration of Helsinki.

### 2.2. Study Sample

This study group comprised all patients with glaucoma during the period 1997 to 2010. Topical glaucoma medications included PGAs, *β*-blockers, *α*-agonists, CAIs, cholinergic agonist, *β*-blockers/CAIs fixed combinations, *β*-blockers/*α*-agonists fixed combination, *β*-blockers/PGAs fixed combinations, and adrenergic agonists. Data on frequency of trabeculectomy were also collected.

### 2.3. Statistical Analyses

The annual expenditures for glaucoma patients were adjusted for inflation as of 2011. The trend test for expenditure was analyzed with linear regression weighted with the inverse of squares residual. The associations between increase and decline in medication costs were analyzed with Pearson correlation. The trend test for frequency of trabeculectomy was analyzed with the Chow test [14]. We also assessed the effect of demographic factors on glaucoma expenditure using a generalized estimating equation regression model (GEE). A *P* value <0.05 was considered to indicate statistical significance; all tests were two-tailed. All statistical analyses were conducted using the statistical package SAS for Windows (Version 9.2).

## 3. Results

The number of beneficiaries included in the LHID2000 sample declined from 916,626 persons in 1997 to 859,913 persons in 2010. The number of patients receiving antiglaucoma drugs increased from 3105 in 1997 to 7033 in 2010 (Table 1).

### 3.1. Overall Costs

**Figure 1:**
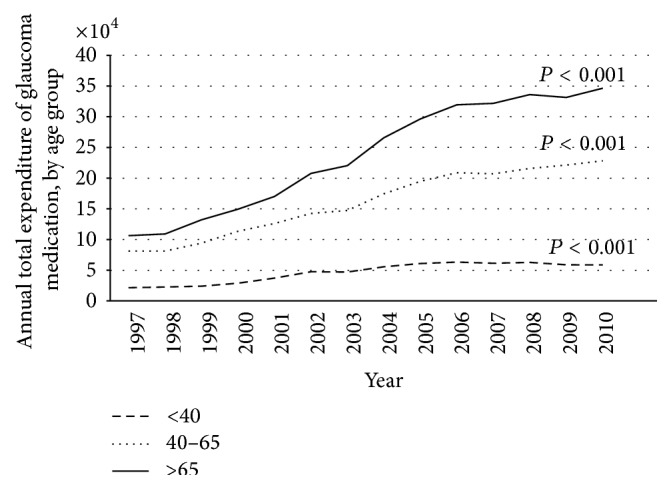
Plot of annual total expenditure of glaucoma medication, by age group.

After adjusting for inflation, the total annual medical expenditures increased from $0.21 million in 1997 to $0.63 million in 2010 (slope = $37,618/year, *P* < 0.001) (Table 1). The increase in total expenditures was remarkable in both genders, every age group, every income group, and every occupation group (*P* < 0.05). Annual glaucoma medication expenditures for men were higher than those for women after 2000. The annual glaucoma medication expenditures were most prominent in the age group ≥65 years, followed by the age group 40–64 years and the age group <40 years (Figure 1). The lowest income group spent more money on antiglaucoma medications than other income groups. The white-collar occupation group spent more money on medications than the other occupation groups.

### 3.2. Per Capita Expenditure Costs

The mean medication expenditure per person increased from $67.3 in 1997 to $90 in 2010 after adjusting for inflation (slope = $2.8/year, *P* < 0.001) (Table 1). The mean cost per capita increased year on year from 1997 to 2006 and then decreased gradually after 2007. The trend in mean medication expenditures per person was similar in each category (gender, age, income, and occupation group). Estimates from the GEE conducted to determine demographic variables associated with the increase in glaucoma medication expenditure indicate that patients ≥40 years incurred higher costs for medication than patients under the age of 40 years (*P* < 0.001). In the same analysis, men incurred higher costs than women (*P* < 0.05), populations with higher income incurred higher costs for medications than populations with lower income (*P* < 0.05), glaucoma medication expenditures increased year on year (*P* < 0.001), and blue-collar workers had lower expenditures than the other types of workers (*P* < 0.001) (Table 2).

### 3.3. The Changes of Expenditure between Different Glaucoma Medications

**Figure 2:**
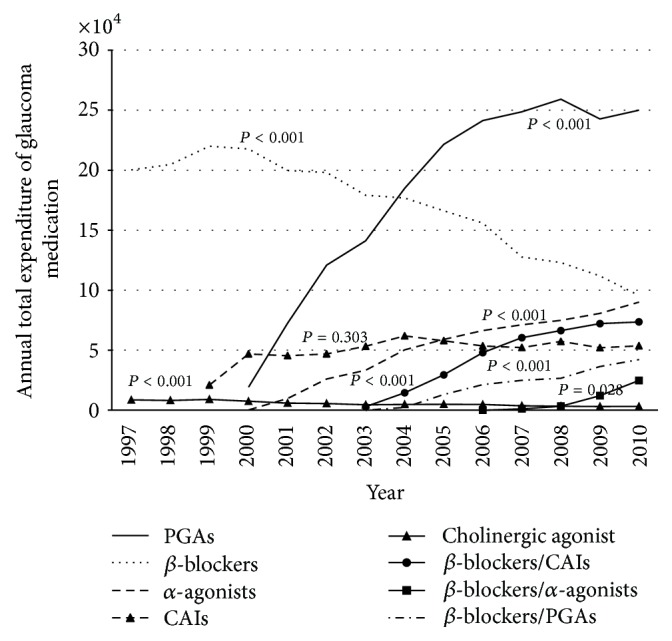
Plot of total expenditure of glaucoma medication, by medication class.

The annual expenditures for most classes of glaucoma medications increased during the study period, except for *β*-blockers (slope = −$10,152/year, *P* < 0.001) and cholinergic agonists (slope = −$432/year, *P* < 0.001) (Table 1). The decrease in expenditure for *β*-blockers was associated with the administration of PGAs (*P* = 0.002), and *α*-agonists (*P* < 0.001). A significant increase in expenditures on PGAs (slope = $23,779/year, *P* < 0.001) was also noted. Medications containing PGAs accounted for 46% of the total glaucoma medication expenditure in 2010 (Table 1) (Figure 2).

We also found a decreasing trend in per capita medication expenditures for *β*-blockers (slope = −$0.5/year, *P* < 0.001) and cholinergic agonists (slope = −$0.5/year, *P* < 0.001). However, the trends for other glaucoma medications were not significant (Table 1).

### 3.4. The Change of Trabeculectomy Number during the Study Period

The numbers of trabeculectomies performed during the study period are shown in Table 1. During the period 1997–2010, we found that the frequency of trabeculectomy had two break points, one in the year 1999 and the other in the year 2000 based on the Chow test [14].

## 4. Discussion

During the study period, the total expenditures for glaucoma medications significantly increased by 3.03-fold because of an increase in patient numbers and an increase in mean medication expenditures per person. The increase in patient numbers may be related to the early diagnosis due to advanced diagnostic modalities (e.g., optical coherence tomography), more accurate diagnosis, overdiagnosis, aging population, or good medical accessibility in Taiwan [10, 11, 15]. The increase in mean medication expenditure per person may be associated with the administration of PGAs and more aggressive glaucoma treatment [5]. The rising cost of glaucoma drugs after PGAs launching also occurred in Ireland, Scotland, Australia, Denmark, and France [16].

As seen in Table 2, mean glaucoma medication expenditures increased markedly after the NHI system began covering treatment with PGAs in 2000. The reduction of expenditure per capita after 2007 and the mean medication cost in Taiwan was much lower than in Denmark and the United States [8, 9] reflecting the success of bargaining medication cost and prescription policy (beta-blocker should be first-line drug) by Taiwan NHI. However, the increasing total medication expenditure imaged that the cost down policy could not offset the growing medical demand.

In this study, expenditures for glaucoma medications were significantly higher for men than for women, whereas in the USA the opposite is true [8]. The possible reasons are differences in knowledge of health issues between genders in different societies and the higher prevalence of ACG in Taiwan [2, 17]. Women are at higher risk of ACG [18] that can be treated with laser iridotomy or cataract surgery rather than glaucoma medications. Cataract surgery is readily available in Taiwan and the procedure reduces the likelihood of developing the disease [13, 19].

It is not surprising that glaucoma medical expenditures were highest among patients ≥65 years because aging is one of the risk factors for developing glaucoma. In this study, the glaucoma medical expenditures for the age group over 65 years significantly increased and accounted for more than half the amount of medical expenditures; a reflection of Taiwan's aging population has great financial burden in glaucoma care. Furthermore, we also found that expenditures for glaucoma medications increased significantly for patients in the age group <40 years, possibly because of advanced diagnostic tools, more aggressive glaucoma treatment, and the high prevalence of myopia among younger people in Taiwan [5, 15, 20].

The study results show that blue-collar workers have lower glaucoma medical expenditures, while higher income families incur higher glaucoma medical expenditures, indicating inequality in health care services under universal health coverage in Taiwan. Such inequality may be the result of differences in general knowledge of health care, out-of-pocket payment policy, and other socioeconomic disparities [10, 21, 22].

The total expenditure for *β*-blockers decreased because of the reduction in cost by the bureau of NHI and the decrease in usage of *β*-blockers, mainly due to the increased availability of PGAs and *α*-agonists. However, considering the drug price, the Taiwan NHI stipulates that *β*-blockers are first-line medications and PGAs, CAIs, and fixed combinations are second-line medications. Therefore, *β*-blockers still have a considerable market share in Taiwan, despite the fact that PGAs are more effective, are associated with fewer adverse effects, require only once-a-day dosing, and are associated with greater patient compliance [23]. The medical expenditures in the USA have also undergone similar changes relative to insurance coverage [8].

Cholinergic agonists are mainly used for ACG. Even though most people in Taiwan are Chinese in origin and have a higher prevalence of ACG [2, 17], there has been a decreasing trend in the application of cholinergic agonists, which may be offset by the increased availability of other drugs like PGAs and the increase in frequency of cataract surgery in Taiwan [13, 23]. The trend in increasing expenditures for fixed combinations of medications during the period of study can be attributed to patients' preference, because the fixed combinations improve medical adherence and reduce eye discomfort [6].

Trabeculectomy is the most common glaucoma surgery in Taiwan. Our study reveals that the number of trabeculectomies decreased significantly in 1999 and 2000, at the time when PGAs were launched and the Taiwan NHI began to reimburse expenses for PGAs. A similar situation of decreasing the number of trabeculectomies after PGAs listing was also reported in Scotland, France, and Australia [16]. Trabeculectomy is more effective in reducing IOP and lowering diurnal tension than PGAs [24]. However, the side effects of trabeculectomy are greater than those associated with PGAs. According to Cutler and McClellan [25], technological change affects treatment in two ways—treatment substitution and treatment expansion or both. Treatment substitution implies a new technology in place of an old one. Treatment expansion takes place when treatments become safer and easier, and patients pay more attention to their conditions when therapy is more effective or less side effective. Based on the results of this study, we presume that PGAs may have the effect of treatment expansion for trabeculectomy under universal health coverage.

There are several limitations to this study. First of all, the study ignored patient adherence and disease severity. Poor patient adherence may reduce medication expenditures at first but eventually will increase medical and surgical expenditures due to symptom complications. Realistically, severities of glaucoma are related to medical expenditures. Secondarily, insured salary is not necessarily truly representative of patients' socioeconomic status. Most people are employees and pay the insurance fee according to their salary. However, the health insurance fee is lower for those living on their investments. We cannot definitely determine the interaction between socioeconomic status and glaucoma medical expenditures. Thirdly, we did not characterize which types of glaucoma and which education levels contributed to medical expenditures.

## 5. Conclusions

This nationwide population-based study demonstrated an increasing trend in glaucoma medical expenditures from 1997 to 2010 in Taiwan. The main factors contributing to these trends include administration of brand-name drug products such as PGAs, the increasing glaucoma population, good medical accessibility, and possibly more aggressive treatment. PGAs may have the effect of treatment expansion for trabeculectomy. Expenditures were highest among men, patients over 40 years of age, and patients with higher incomes and were lowest among blue-collar workers. The inequality of health care in different socioeconomic disparities may still exist in Taiwan.

## Figures and Tables

**Table 1:** Total and mean glaucoma medication expenditure (USD, adjusted for inflation to the 2011 Consumer Price Index of Taiwan), LHID2000.

	Total glaucoma medication cost (mean cost per person)															
Characteristic	1997	1998	1999	2000	2001	2002	2003	2004	2005	2006	2007	2008	2009	2010	Slope	*P* value
	(*n* ^*∗*^ = 3105)	(*n* = 3503)	(*n* = 3662)	(*n* = 3689)	(*n* = 3824)	(*n* = 4118)	(*n* = 4419)	(*n* = 5049)	(*n* = 5410)	(*n* = 5633)	(*n* = 5932)	(*n* = 6298)	(*n* = 6745)	(*n* = 7033)		
Total expenditure	208,858	212849	249,959	291,523	333,204	397,456	414,489	495,927	552,024	591,417	589,885	614,044	611,331	633,151	37,618	<0.001
	(67.3)	(60.8)	(68.3)	(79.0)	(87.1)	(96.5)	(93.8)	(98.2)	(102.0)	(105.0)	(99.4)	(97.5)	(90.6)	(90.0)	2.8	<0.001
Gender																
Male	100,245	104,485	123,542	147,789	167,646	202,446	214,111	258,024	285,850	307,655	312,891	325,681	325,892	337,195	20,567	<0.001
	(68.1)	(62.7)	(70.9)	(84.1)	(90.6)	(99.9)	(100.1)	(105.5)	(108.3)	(111.0)	(109.1)	(105.0)	(97.9)	(95.7)	3.5	<0.001
Female	108,613	108,364	126,417	143,734	165,558	195,010	200,379	237,903	266,174	283,762	276,994	288,364	285,439	295,956	17,013	<0.001
	(66.6)	(59.0)	(65.9)	(74.4)	(83.9)	(93.3)	(87.8)	(91.4)	(96.1)	(99.1)	(90.4)	(90.2)	(83.6)	(84.3)	2.2	0.004
Age																
<40	21,558	22,522	23,960	28,747	37,071	47,405	46,926	55,595	60,910	63,110	61,386	62,593	58,693	58,665	3,965	<0.001
	(46.3)	(40.1)	(42.2)	(49.5)	(66.8)	(74.5)	(71.8)	(71.4)	(74.1)	(80.2)	(77.6)	(74.8)	(67.9)	(67.5)	2.6	0.001
40–64	81,136	81,205	93,961	113,495	126,037	142,456	147,223	174,562	194,788	208,877	206,852	215,594	221,214	228,075	12,963	<0.001
	(64.7)	(59.6)	(65.8)	(78.1)	(83.6)	(90.8)	(85.0)	(89.2)	(93.5)	(95.9)	(90.5)	(89.5)	(83.9)	(84.3)	2.0	0.003
≥65	106,165	109,121	132,038	149,281	170,096	207,595	220,340	265,770	296,327	319,430	321,647	335,858	331,424	346,410	20,982	<0.001
	(76.7)	(69.1)	(79.3)	(90.2)	(96.6)	(108.5)	(108.4)	(114.9)	(118.3)	(119.8)	(112.6)	(110.0)	(102.2)	(100.2)	3.3	<0.001
Monthly income																
≤610	121,072	117,866	135,830	158,382	183,680	216,500	225,130	263,846	287,919	300,211	291,968	299,156	288,696	292,996	17,296	<0.001
	(73.7)	(65.6)	(71.6)	(84.7)	(94.1)	(102.7)	(100.1)	(103.8)	(108.0)	(109.4)	(102.9)	(100.6)	(92.3)	(92.2)	2.5	0.005
611–1220	71,896	77,079	92,189	102,596	114,674	137,747	142,387	172,602	194,157	211,463	214,992	228,099	232,210	242,717	14,528	<0.001
	(58.9)	(55.2)	(64.5)	(71.0)	(76.3)	(85.2)	(82.3)	(88.5)	(90.5)	(95.1)	(91.0)	(91.0)	(85.1)	(84.3)	2.9	<0.001
>1220	15,889	17,904	21,940	30,546	34,850	43,209	46,972	59,480	69,948	79,743	82,926	86,789	90,426	97,439	6,867	<0.001
	(65.7)	(57.9)	(65.3)	(82.1)	(94.2)	(109.7)	(107.0)	(106.8)	(117.0)	(119.9)	(113.1)	(106.2)	(102.1)	(99.7)	4.0	0.001
Occupation																
White-collar	90,794	92,736	108,883	127,199	147,618	172,884	176,803	215,931	248,346	268,507	273,476	287,715	289,024	303,343	18,495	<0.001
	(69.5)	(62.4)	(69.0)	(80.2)	(88.3)	(95.9)	(90.7)	(93.5)	(100.2)	(103.6)	(99.8)	(96.5)	(90.3)	(90.1)	2.5	0.001
Blue-collar	67,431	69,645	81,244	91,903	102,067	123,503	128,240	153,700	172,262	188,362	188,384	196,023	195,304	205,480	11,985	<0.001
	(58.7)	(53.2)	(60.8)	(68.1)	(74.3)	(83.4)	(81.6)	(86.7)	(88.9)	(93.0)	(88.1)	(87.2)	(81.3)	(82.1)	2.7	<0.001
Other	50,633	50,468	59,832	72,422	83,519	101,069	109,446	126,296	131,416	134,548	128,025	130,307	127,004	124,328	7,902	<0.001
	(78.0)	(71.4)	(79.9)	(96.2)	(107.4)	(120.9)	(121.9)	(130.6)	(132.2)	(132.4)	(121.6)	(122.1)	(111.1)	(107.0)	4.0	0.002
Glaucoma medication^a^																
PGAs				19,283	72,406	120,877	141,191	185,021	221,495	241,386	248,635	259,079	242,699	250,057	23,779	<0.001
				(80.0)	(138.2)	(150.2)	(127.0)	(127.0)	(132.8)	(135.2)	(130.9)	(129.2)	(116.5)	(112.6)	0.4	0.787
*β*-blockers	200,229	204,690	219,951	217,671	199,730	198,074	179,209	176,870	166,256	155,935	127,751	123,041	111,916	95,682	−10,152	<0.001
	(67.3)	(60.4)	(62.1)	(61.4)	(57.1)	(56.1)	(50.3)	(46.3)	(43.9)	(41.5)	(34.5)	(32.2)	(28.2)	(24.3)	−0.5	<0.001
*α*-agonists				180	9,720	26,053	33,311	50,288	58,937	66,428	71,139	74,891	80,729	90,018	8,697	<0.001
				(18.0)	(37.4)	(52.6)	(54.3)	(54.8)	(54.5)	(55.2)	(52.2)	(50.5)	(48.0)	(48.4)	1.5	0.194
CAIs			21,002	46,907	45,410	46,897	53,271	61,980	57,895	53,574	52,206	57,326	52,120	53,699	648	0.303
			(72.2)	(92.9)	(83.0)	(83.0)	(84.3)	(91.8)	(90.0)	(85.4)	(81.1)	(84.9)	(75.0)	(70.9)	−1.5	0.060
Cholinergic agonist	8,629	8,159	9,005	7,483	5,939	5,554	4,530	4,863	4,978	4,751	3,747	3,331	3,138	3,166	−432	<0.001
	(10.9)	(9.5)	(11.5)	(10.5)	(9.8)	(9.4)	(8.0)	(8.2)	(8.4)	(8.1)	(6.9)	(6.3)	(5.9)	(5.9)	−0.5	<0.001
*β*-blockers/ CAIs							2,951	14,553	29,437	47,992	60,413	66,376	72,166	73,586	11,526	<0.001
							(57.9)	(77.8)	(87.3)	(93.2)	(99.2)	(93.1)	(95.7)	(90.4)	3.0	0.072
*β*-blockers/ *α*-agonists										34	1,134	3,373	12,115	24,760	5,750	0.028
										(17.1)	(51.5)	(54.4)	(64.8)	(79.9)	12.6	0.076
*β*-blockers/ PGAs							26	2,351	13,026	21,316	24,860	26,603	36,433	42,184	8,042	<0.001
							(25.8)	(58.8)	(105.9)	(134.9)	(146.2)	(116.2)	(121.4)	(114.9)	9.8	0.079
Number of trabeculectomies	124	126	100	69	73	65	64	66	63	70	75	61	70	68		

PGAs, prostaglandin analogues; CAIs, carbonic anhydrase inhibitors; LHID2000, National Longitudinal Health Insurance Database 2000.

*n* ^*∗*^, number of patients under glaucoma medication.

^a^Adrenergic agonists not shown because of small sample sizes.

**Table 2:** The results of generalized estimating equations regression model (USD/year).

	Estimate	SE	*P* value
Intercept	33.4	1.5	<0.001
Age, year (versus <40)			
40–64	10.4	1.4	*<*0.001
≥65	18.5	1.5	<0.001
Gender (versus female)	2.2	1.1	0.041
Income, USD (versus ≤610)			
611–1220	−3.2	1.3	0.015
>1220	6.7	2.0	<0.001
Occupation (versus white-collar workers)			
Blue-collar workers	−6.7	1.3	<0.001
Other types of workers	6.8	1.6	<0.001
Year	1.0	0.1	<0.001
